# Stable stomatal number per minor vein length indicates the coordination between leaf water supply and demand in three leguminous species

**DOI:** 10.1038/s41598-017-02448-y

**Published:** 2017-05-19

**Authors:** Wan-Li Zhao, Zafar Siddiq, Pei-Li Fu, Jiao-Lin Zhang, Kun-Fang Cao

**Affiliations:** 1 Key Laboratory of Tropical Forest Ecology, Xishuangbanna Tropical Botanical Garden, Chinese Academy of Sciences, Menglun, Mengla, Yunnan Province 666303 China; 2 School of Life Sciences, University of Science and Technology of China, Hefei, Anhui Province 230026 China; 3 Key Laboratory of Vegetation Restoration and Management of Degraded Ecosystems, South China Botanical Garden, Chinese Academy of Sciences, Guangdong Province, 510650 China; 4 Plant Ecophysiology and Evolution Group, State Key Laboratory for Conservation and Utilization of Subtropical Agro-Bioresources, and College of Forestry, Guangxi University, Nanning, Guangxi 530004 China

## Abstract

The coordination between minor vein density (MVD) and stomatal density (SD) has been found in many plants. However, we still know little about the influence of leaf node on this correlation relationship. Here, we devised the new functional trait ‘stomatal number per minor vein length’ (SV). By measuring leaflet area (LA), MVD, SD, and SV, we demonstrated the significance of this functional trait in *Arachis hypogaea* (peanut) grown under different light regimes and in sun leaves of *Dalbergia odorifera* and *Desmodium renifolium*. We found that SV did not change significantly with leaflet node or with LA within each light treatment, while shading caused a significant decrease in SV. The positive correlation between SD and MVD was found in peanut under each light regime. Sun leaves of *D. odorifera* and *D. renifolium* also had stable SV along the leaflet node, with a positive correlation between MVD and SD. We conclude that under a certain light regime, a stable SV similar to the positive correlation between MVD and SD can also indicate the coordination between leaf water supply and demand. Our findings highlight the significance of SV and provide new insight into the coordination between stomatal number and minor vein length.

## Introduction

Leaf veins provide a pathway of low resistance for water flow through the mesophyll tissue to evaporative surfaces near the stomata where it is exchanged for CO_2_^1, 2^. Indeed, minor vein density (MVD) has been shown to act as a principal determinant of leaf hydraulic supply capacity across land plant diversity^3^. The exchange of water vapour and CO_2_ between leaf tissues and the atmosphere mainly occur at the site of stomata^4^, thus stomatal density (SD) and stomatal size could dictate maximum transpiration and therefore leaf water demand^5^. Increasing the investment in vein plumbing can support greater photosynthetic capacity because more water can be delivered to the sites of transpiration, thus maintaining optimal stomatal aperture for greater exchange of CO_2_^5, 6^. Therefore, maintaining an optimal coordination between MVD and SD during adaptation to the environment should be of highly functional and adaptive importance for plants^1, 7^. Until now, many studies have found a positive correlation between MVD and SD in different plant species^1, 7–10^. However, any responsible functional trait for this coordination is still relatively unknown.

As the coordination between MVD and SD can be seen as a status of plant under certain environmental conditions, which may be disturbed with changing ambient environment^10, 11^. In previous studies, the correlation between MVD and SD was characterized as strong positively^1^, weak positively^10^, or marginal^12^. However, few studies compared the differences in their correlation^10, 11^. By comparing the slopes of positive correlation between SD and MVD, Carins Murphy *et al*.^11, 13^ found that their coordination in *Toona ciliata* M. Roem. did not change in the leaves produced by plants acclimated to different vapour pressure and irradiance treatments. While other studies have reported an inconsistent pattern between MVD and SD^10, 14^. Torre *et al*.^14^ found that Baroness roses had decreasing SD with an increase in MVD with increasing vapor pressure deficit (VPD). Having excess venation will be inefficient due to both the high carbon cost of synthesizing lignin (the main component of leaf venation)^15^ and the loss of photosynthetic potential resulting from the displacement of photosynthetic tissue by vascular tissue^11^. However, aridity-adapted *Eucalyptus* and *Corymbia* species have apparent over-investment in leaf vein density to offset the negative effect of leaf thickness on photosynthesis^16^. As MVD or SD responds to environmental changes in species-specific ways, the assessment in the change of their coordination can indicate the responses of species to varying ambient environmental conditions, and there may be the other functional trait governing their coordination.

Previous studies have reported some indexes obtained from the ratio of other two indexes, for example, leaf mass per area (LMA)^17^ and stem hydraulic conductivity per sapwood area (sapwood specific hydraulic conductivity, *K*_s_)^18^, which simply explain some physiological processes (for example, LMA as the leaf-level cost of light interception^17^). In this study, we propose a new functional trait: the stomatal number per minor vein length (SV, no. mm^−1^), which is calculated by dividing SD (no. mm^−2^) by MVD (mm. mm^−2^). Compared to the correlation between MVD and SD, SV as a parameter of functional trait just excludes the area component. The SV reflects the coordination between transpiration of a given number of stomata and water supplied per unit length of minor veins. In other words, the water that is supplied per unit of minor vein length could meet the demand of a given number of stomata. Although the distance from the vein to the stomata appears to be the key component in determining leaf hydraulic conductivity^3^, and the free-ending veinlets as the final order of venation, play a major role in homogenizing this distance^19^. However, just like the positive correlation between MVD and SD, which was first found in *Nothofagus cunninghamii* (Hook.) Oerst. trees^1^, SV is calculated by dividing SD by MVD and also has little relation to the distance from the vein to the stomata. Although both SV and the slope of the linear regression between SD and MVD (SD = b + a × MVD) can indicate the proportional change in stomatal number with minor vein length, they have different biological significance and could have the same numerical value only when the intercept of the linear regression is zero^19^. At present, studies comparing the differences between SV and MVD and SD are quite rare.

Variations in light regime can have a strong influence on both MVD and SD^1, 20, 21^. Some species’ leaves are typically smaller and thicker when exposed to full sun light than those grown in the shade. Such sun exposed leaves can have greater MVD and SD^13, 20, 22^. These studies have documented the effect of shading on MVD and SD, however, little is known about the influence of extreme low light on changes in their coordination. Although it is rare to have direct evidence for coordinated vein and stomatal development through the leaf expansion phase^13^ for herbaceous plants, venation density and SD increase with increasing leaf insertion level^23–25^. In *Dendrobium* species, the MVD was independent of leaf area but SD was significantly and negatively correlated with leaf area^26^. However, MVD was strongly and negatively correlated with leaf area among populations of *N. cunninghamii* grown in different environmental conditions^1^. Although many studies have reported the relationship between leaf area and SD and MVD^1, 10, 11, 13, 26^, yet little is known about the relationship between leaf area and SV.

*Arachis hypogaea* L. (peanut) is an important oilseed legume around the world; it can be easily planted and grows quickly. Peanut can produce a series of differently shaped and sized leaflets along the leaf node of a stem. Its leaflets do not have trichomes and the minor veins and stomata can be easily imaged. For these reasons, we chose peanut as our study model and assumed that peanut plants grown in different light regimes could provide us with insights into the coordination between minor veins and stomata. Under the similar environmental conditions, to support the water demand of the same number of stomata, we would expect leaves to develop the same or similar length of minor veins, independent to the leaf size and shape, which will make the most efficient investment in leaf xylem relative to photosynthetic gain^1^. We hypothesized that (1) the SV remained stable under the same light regime, indicating the coordination between leaf water supply and demand in peanut plants, (2) the SV would be changed when the changing ambient environment caused the difference on this coordination, and (3) the SV was uncorrelated with leaflet area. We also used sun leaves of *Dalbergia odorifera* T. Chen and *Desmodium renifolium* (L.) Schindl. to test our first and third hypotheses. Peanut and the selected two species belong to Leguminosae, representing an herb with compound leaves (peanut), a tree with compound leaves (*D. odorifera*), and a shrub with a unifoliolate leaf (*D. renifolium*).

## Results

**Figure 1:**
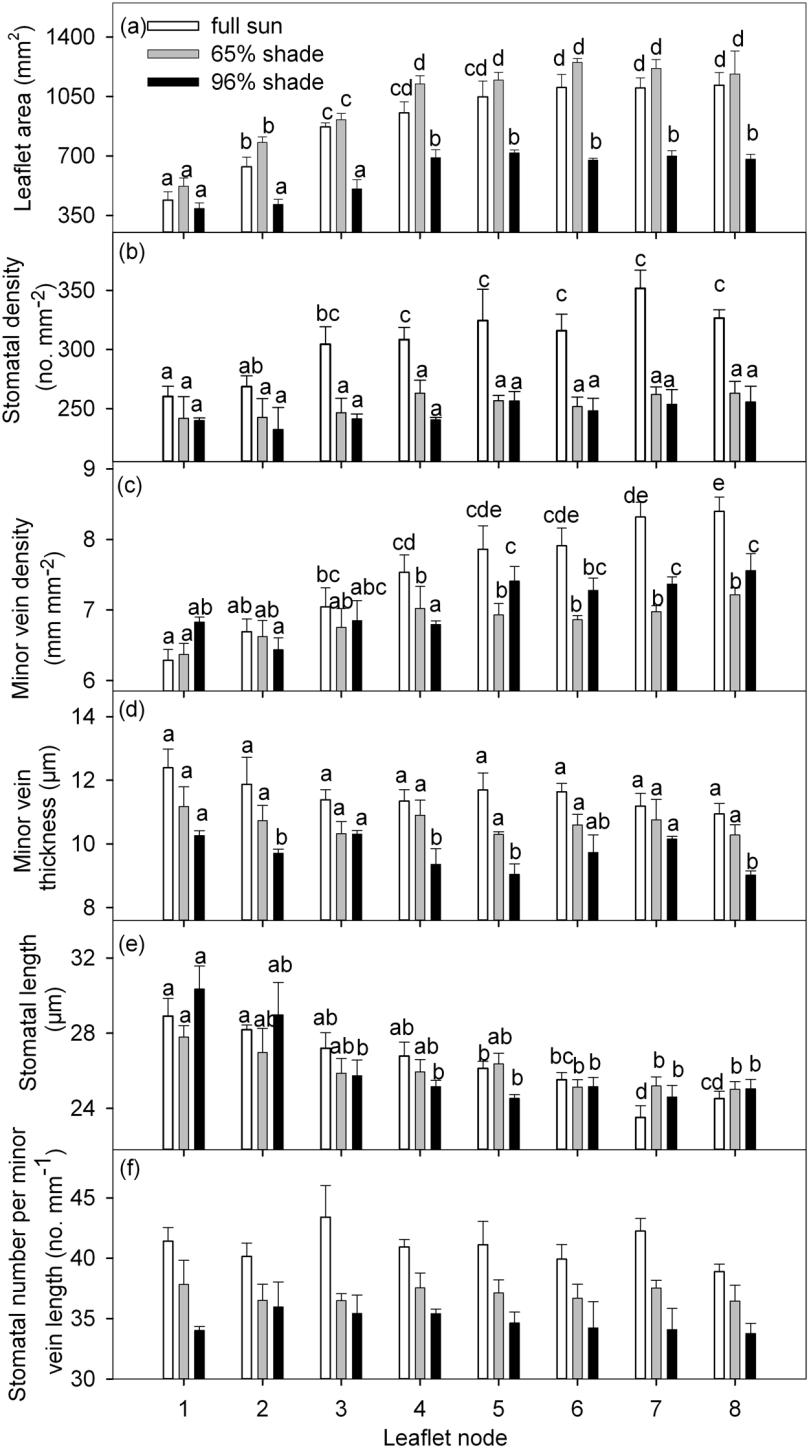
Leaflet area (**a**), stomatal density (**b**), minor vein density (**c**), minor vein thickness (**d**), stomatal length (**e**) and stomatal number per minor vein length (SV, **f**) of the apical leaflet from the first to eighth leaf in node on peanut plants grown in three light conditions. Different lowercase letters in each panel indicate a significant difference in the traits of plants in the same light condition (*P* < 0.05). Under each light condition, SV did not significantly change with the leaf node (**f**).

Both LA and MVD increased with leaflet node from 1^st^ to 4^th^ within each of the three light regimes, while no significant changes with leaflet node from 5^th^ to 8^th^ (Fig. 1a,c). SD of the apical leaflet also increased from the 1^st^ to 3^rd^ leaflet node under the full sun treatment, but it remained relatively unchanged under the two shading treatments (Fig. 1b). Minor vein thickness (MVT) decreased with leaflet node (from 1^st^ to 8^th^) within each of the three light regimes (Fig. 1d). The SL decreased with leaflet node from 1^st^ to 4^th^ within each of the three light regimes, while there was no significant changes with leaflet node from 5^th^ to 8^th^ (Fig. 1e). SV ranged from 38.9–43.4 mm^−1^, 36.5–37.8 mm^−1^ and 33.8–36.0 mm^−1^ under full sun, 65% shade and 96% shade, respectively (Fig. 1f), and did not change significantly with respect to leaflet nodes within each light treatment (Fig. 1f), but it decreased with decreasing light levels (Tables 1 and 2).

**Table 1:** Leaflet traits (mean ± SE) of peanut plants grown under three different light intensities.

Trait	Unit	full sun	65% shade	96% shade
LA	mm^2^	908.8 ± 88.1^a^	1016.9 ± 90.9^a^	597.3 ± 48.4^b^
MVD	mm mm^−2^	7.5 ± 0.3^a^	6.8 ± 0.1^b^	7.1 ± 0.1^ab^
MVT	μm	11.6 ± 0.2^a^	10.6 ± 0.2^b^	9.7 ± 0.1^c^
SD	no.mm^−2^	307.5 ± 10.7^a^	253.4 ± 3.2^b^	246.0 ± 3.1^b^
SL	μm	25.9 ± 0.5^a^	26.0 ± 0.4^a^	26.2 ± 0.4^a^
SV	no.mm^−1^	41.0 ± 0.5^a^	37.0 ± 0.2^b^	34.7 ± 0.3^c^

LA = leaflet area, MVD = minor vein density, MVT = minor vein thickness, SD = stomatal density, SL = stomatal length, SV = stomata number per minor vein length. Different letters indicate a significant difference in a leaf trait between different light conditions (*P < *0.05).

**Table 2:** Results of two-way ANOVA to test for the effect of light intensity and node on leaflet traits in peanut.

	D.F.	F	*P*
Leaflet area (mm^2^)			
Position on the stem	7	21.8	***
Illumination treatment	2	61.6	***
Error	14		
Stomatal density (no. mm^−2^)			
Position on the stem	7	3.7	*
Illumination treatment	2	47.4	***
Error	14		
Minor vein density (mm mm^−2^)			
Position on the stem	7	6.0	**
Illumination treatment	2	9.0	**
Error	14		
Stomatal number per minor vein length (no. mm^−1^)			
Position on the stem	7	1.5	ns
Illumination treatment	2	96.8	***
Error	14		
Stomatal length (μm)			
Position on the stem	7	9.3	***
Illumination treatment	2	0.2	ns
Error	14		
Minor vein thickness (μm)			
Position on the stem	7	3.3	*
Illumination treatment	2	63.3	***
Error	14		

D.F. degrees of freedom. *P < 0.05; **P < 0.01;

***P < 0.001; ns, *P* > 0.05.

**Figure 2:**
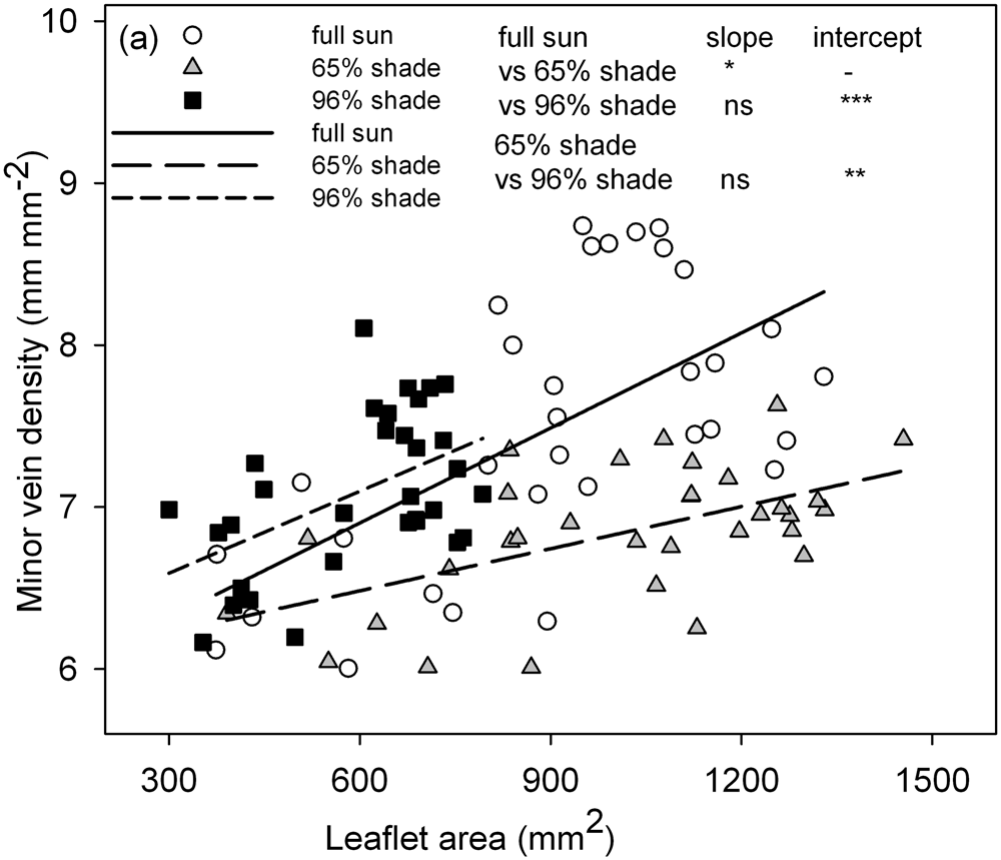
Relationships between leaflet area (LA) and minor vein density (MVD) in peanut plants grown in three light conditions. Each symbol represents one leaflet, and relationships were significant for each light condition (full sun, MVD = 5.7 + 0.002 × LA, *r* ^2^ = 0.38***; 65% shade, MVD = 6.0 + 0.001 × LA, *r* ^2^ = 0.31**; 96% shade, MVD = 6.1 + 0.002 × LA, *r* ^2^ = 0.27**). The differences in linear regression slope and intercept between each pair of lines are indicated. *P < 0.05; **P < 0.01; ***P < 0.001; ns, *P* > 0.05.

**Figure 3:**
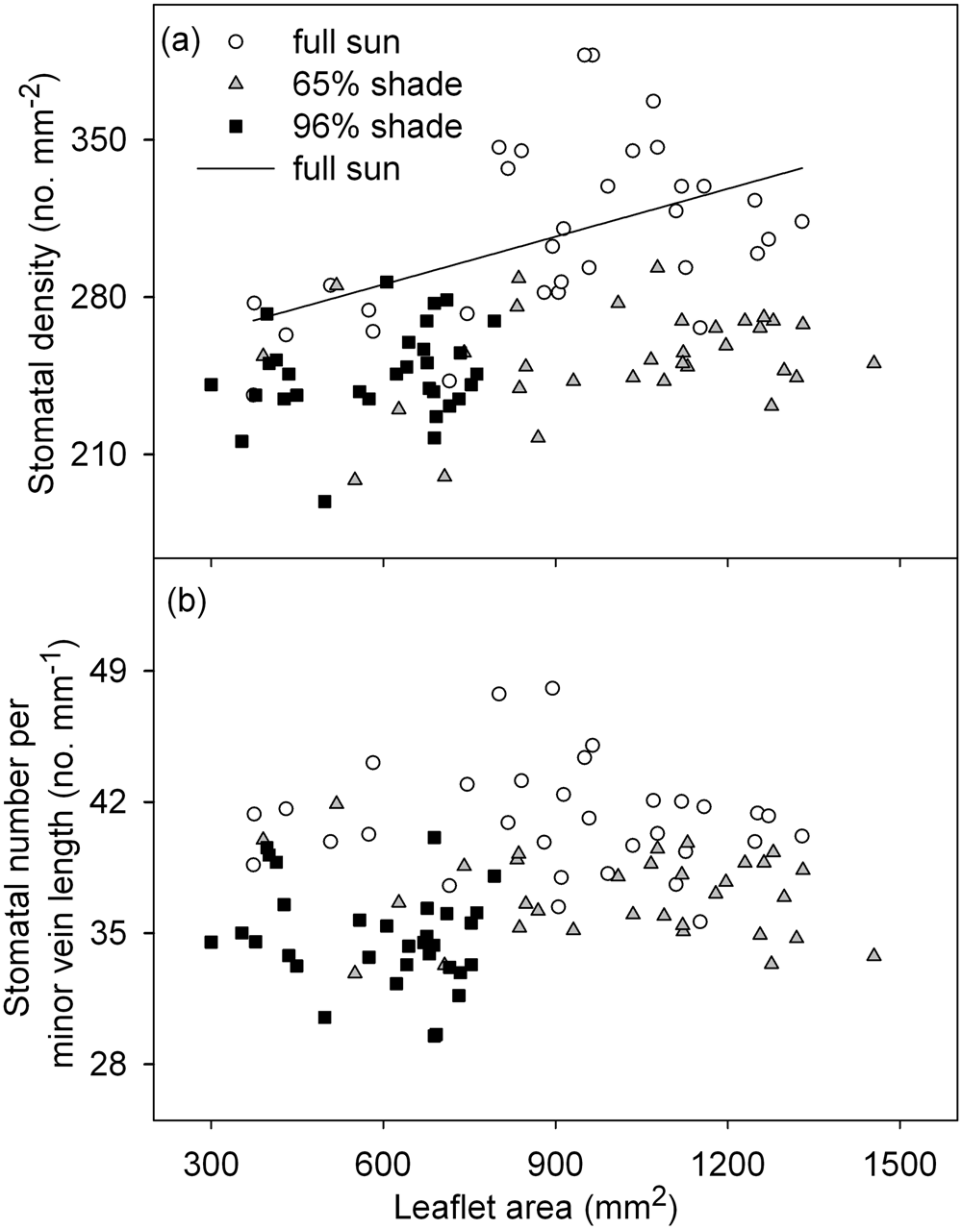
Relationships between leaflet area and stomatal density (**a**) and stomata number per minor vein length (**b**) for peanut plants grown in three light conditions. Each symbol represents one leaflet. The relationship between leaflet area and stomatal density was significant in full sun condition (SD = 243.1 + 0.07 × LA, *r* ^2^ = 0.24, *P < *0.01).

The LA was significantly and positively correlated with MVD in peanut plants within each light condition (full sun, MVD = 5.7 + 0.002 × LA, *r* ^2^ = 0.38, *P < *0.001; 65% shade, MVD = 6.0 + 0.001 × LA, *r* ^2^ = 0.31, *P < *0.01; 96% shade, MVD = 6.1 + 0.002 × LA, *r* ^2^ = 0.27, *P < *0.01; Fig. 2). In contrast, the peanut plants in 96% shade had significantly higher regression intercept than those from the full sun and 65% shading. The LA was significantly and positively correlated with SD in the full sun condition (SD = 243.1 + 0.07 × LA, *r* ^2^ = 0.24, *P < *0.01; Fig. 3a), but there was no coordination in the two shade treatments. The SV was not correlated with LA in any of the light treatments (Fig. 3b).

**Figure 4:**
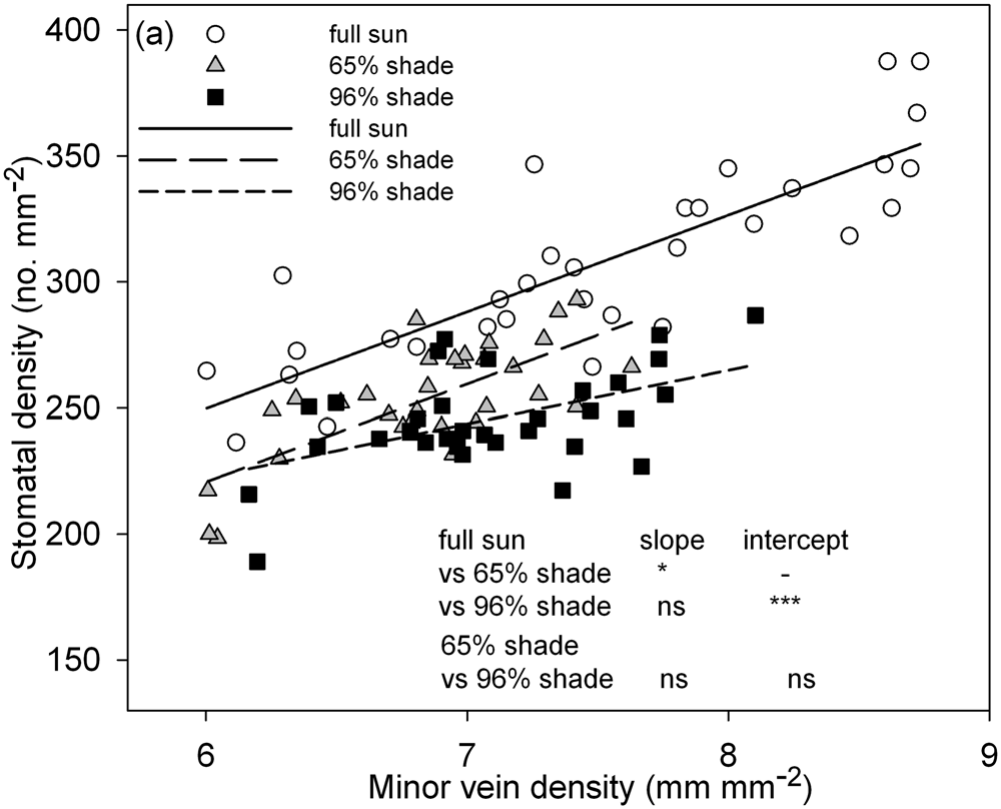
Relationships between minor vein density (MVD) and stomatal density (SD) for peanut plants in three light conditions. Each symbol represents one leaflet, and relationships were significant for each light condition (full sun, SD = 19.4 + 38.4 × MVD, *r* ^2^ = 0.70***; 65% shade, SD = −12.1 + 38.8 × MVD, *r* ^2^ = 0.53***; 96% shade, SD = 95.3 + 21.2 × MVD, *r* ^2^ = 0.26**). The differences in linear regression slope and intercept between each pair of lines are indicated. *P < 0.05; **P < 0.01; ***P < 0.001; ns, *P* > 0.05.

A significant and positive correlation between MVD and SD was found within each light treatment (full sun, SD = 19.4 + 38.4 × MVD, *r* ^2^ = 0.7, *P < *0.001; 65% shade, SD = −12.1 + 38.8 × MVD, *r* ^2^ = 0.53, *P < *0.001; 96% shade, SD = 95.3 + 21.2 × MVD, *r* ^2^ = 0.26, *P < *0.01; Fig. 4). The regression slope between MVD and SD in the full sun plants was significantly higher than for those in 65% shade, and the regression intercept for the full sun plants was significantly higher than those in 96% shade. The regression slope and intercept for the plants in 65% shade were not significantly different from those in 96% shade (Fig. 4).

**Figure 5:**
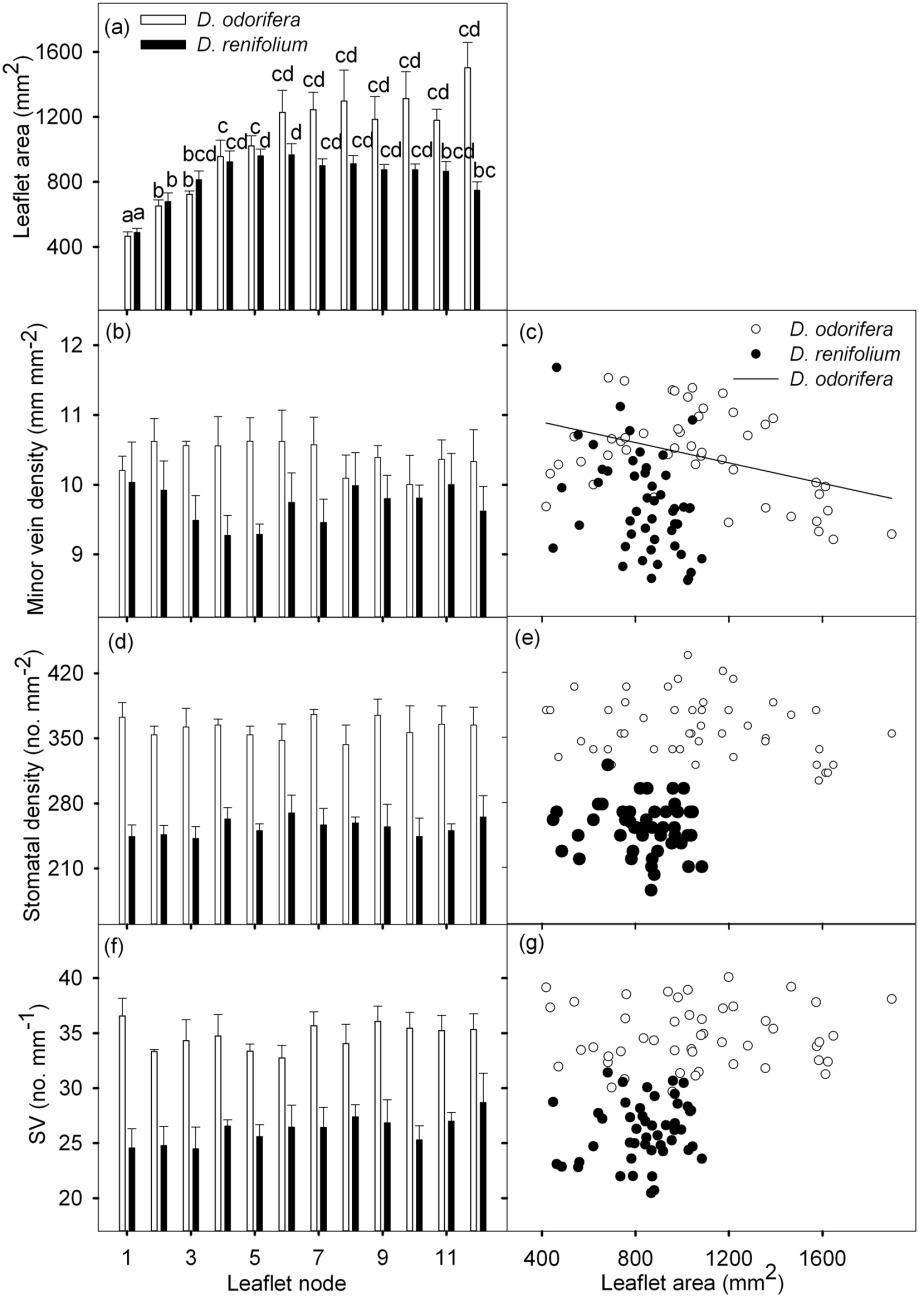
Relationships between traits in sun leaves within *D*. *odorifera* and *D*. *renifolium*. (**a**), (**b**), (**d**) and (**f**) Relationships between leaflet area, minor vein density (MVD), stomatal density (SD), stomatal number per minor vein length (SV), and leaf node (leaflet node for *D*. *odorifera*). Leaflets were numbered according to their order of production on the rachis or on the shoot. Different lowercase letters in each panel indicate a significant difference in the traits of the same species (*P* < 0.05). For *D*. *odorifera* and *D*. *renifolium*, MVD, SD and SV did not significantly change with the leaflet node. (**c**), (**e**) and (**g**) Relationships between leaflet area (LA), MVD (significant for *D*. *odorifera*, MVD = 11.2–0.001 × LA, *r* ^2^ = 0.17, *P* < 0.01), SD, and SV for *D*. *odorifera* and *D*. *renifolium*. Each symbol represents one leaflet.

**Figure 6:**
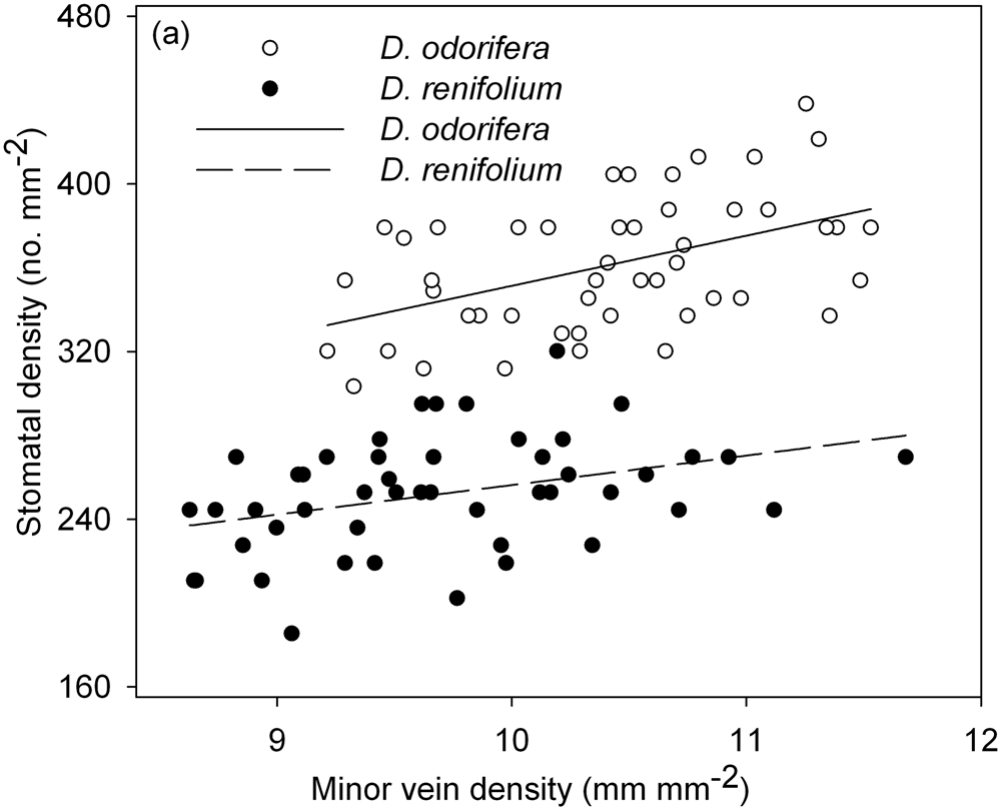
Relationships between minor vein density (MVD) and stomatal density (SD) for *D*. *odorifera* and *D*. *renifolium*. Each symbol represents one leaflet, and relationships were significant for each species (*D*. *odorifera*, SD = 112.2 + 23.9 × MVD, *r* ^2^ = 0.23, *P < *0.001; *D*. *renifolium*, SD = 115.7 + 14.1 × MVD, *r* ^2^ = 0.13, *P < *0.05).

Sun leaflets of *D. odorifera* and leaves of *D. renifolium* also had stable SV along the leaflet node (Fig. 5f) or along the rachis in one leaf (*D. odorifera*), with a positive correlation between MVD and SD (*D. odorifera*, SD = 112.2 + 23.9 × MVD, *r* ^2^ = 0.23, *P < *0.001; *D. renifolium*, SD = 115.7 + 14.1 × MVD, *r* ^2^ = 0.13, *P < *0.05; Fig. 6), although the LA was significantly different along the leaflet node (Fig. 5a). The values of SV of *D. odorifera* and *D. renifolium* sun leaflets ranged from 32.8–36.6 (average 34.7) mm^−1^ and 24.5–28.7 (average 26.1) mm^−1^, respectively (Fig. 5f). The LA of *D. odorifera* was significantly and negatively correlated with MVD (MVD = 11.2–0.001 × LA, *r* ^2^ = 0.17, *P < *0.01; Fig. 5c). The SV was not correlated with LA in sun leaflets of both *D. odorifera* and *D. renifolium* (Fig. 5g).

## Discussion

In this study, we assessed the coordination between stomatal number and minor vein length from two perspectives: (1) the correlation between SD and MVD, and (2) stomatal number per minor vein length (SV) under different light regimes. The positive correlation between SD and MVD under each light regime affirms their coordination in peanut, which also has been reported in the other species^1, 10, 27–29^. As predicted, the leaflets did not exhibit significant differences in SV under each light regime (Fig. 1f, Table 2), suggesting that the change in stomatal number was in proportion to the length of the minor veins. Leaves of *D. odorifera* and *D. renifolium* under full sun conditions also had stable SV along the leaflet node in this study (Fig. 5f). The fact that SV remained stable across light conditions suggests that the coordination between leaf water supply and demand was maintained despite changes in environmental conditions. Similarly merely knowing the values of SD and MVD does not allow one to infer their coordination, while a positive correlation between them allows inferences (Fig. 4). SV links SD and MVD together and may shed light on the coordination between leaf water demand and supply. An advantage of SV is that it can be calculated on a single leaf, whereas the correlation between MVD and SD via regression requires multiple leaves to be estimated.

We found that shading caused significant changes in the correlation between MVD and SD, as revealed by the difference in the regression slope or intercept between them (Fig. 4). Although other factors (humidity, temperature, etc.) under shading conditions may also had influence on minor veins and stomata, due to the smooth air flow through polyethylene screening and the huge difference in light, leaf traits were assumed to be mainly affected by light treatment. Plants that maintain homeostasis in leaf water content should coordinate water supply and demand by maintaining coordination between MVD and SD^30–32^. Compared to tropical mountain forest tree species, in subtropical mountain forest, tree species had lower MVD and higher stomatal length, but lack the significant difference in SD, which was the main reason of clear differences in their coordination between these two types of forests^10^. Compared to peanut plants grown in full sun, the MVD in 65% shade and 96% shade decreased by 9.3% and 5.3%, respectively, while the SD decreased by 17.6% and 20%, respectively. These inconsistent changes in MVD and SD in peanut leaflets in response to sun and shade led to a significant decrease in their correlation after shading (Fig. 4). The SV in peanut plants between each pair of light regimes was significantly different (Table 1), indicating that to meet the water demand of the same number of stomata, the length of minor veins had to be significantly different in sun and shade conditions. Leaf size increases under shade conditions^1, 13^, but extreme low light (96% shade) conditions resulted in LA reduction and provided a new viewpoint to examine changes in the coordination between SD and MVD with LA and SV. After shading, the MVD still had a positive correlation with LA, but there was no significant relationship between SD and LA. As expected, the SV of peanut plants was independent of LA in the studied light treatments.

As both SD and MVD between the two shading treatments showed no significant difference (Table 1), the regression slope and intercept between MVD and SD for the plants grown in 65% shade did not significantly differ from those grown in 96% shade (Fig. 4). This means that their coordination did not significantly change between the 65% shade and the 96% shade treatments. However, the MVT of the plants grown in 65% shade was significantly higher than the MVT of the plants in 96% shade. The coordination between hydraulic supply and demand was not only determined by SD and MVD, but also could be influenced by SL and MVT^1, 7^. The significantly lower MVT of plants in 96% shade than of plants in 65% shade indicates that the shading treatment changed the coordination. However, the measurement of their coordination by the correlation between SD and MVD could not identify this difference. Compared to the plants in the 65% light regime, the lower MVT in 96% shade supported a lower number of stomata per minor vein length, thus leading to the significantly lower SV. Therefore, compared to the differences in the linear regression slope or intercept between MVD and SD, the difference in SV was not only more direct but also more distinct to indicate their coordination change among different light treatments.

Another interesting finding was the positive correlation between LA and MVD of peanut plants within each light regime. This finding contradicts to the negative correlation between MVD and LA reported in other species^1, 23, 33^ and also differs from the independent relationship reported between MVD and leaf size in global ecological patterns of leaf venation architecture^5^. However, the increase in MVD along the leaf insertion node in the peanut plant is consistent with Zalenski’s law, which describes the increasing venation density in grass leaves with insertion height above the ground^25^. Leaf development can be separated into a slow phase (mainly due to cell proliferation) and a rapid phase (mainly due to cell expansion)^2, 34^. Minor veins principally are formed during the rapid phase, and the MVD stabilises as their initiation is maintained during leaf expansion^34^. Changes in vein density may be independent of leaf size if the number of vein orders increases or decreases prior to full leaf expansion^34^. Carins Murphy *et al*.^29^ found that epidermal cell size is a major determinant of MVD and SD in a diverse range of woody and herbaceous angiosperms, while there is no consistent relationship between epidermal cell size and leaf size, which may also explain why MVD is not well correlated with leaf size globally. The higher MVD in larger peanut leaflets may be due to the ongoing vein formation during the expansion phase of leaflet growth. In many species, substantial leaf expansion continues after the formation of vein procambia; therefore, minor vein density declines as leaves continue to expand^23, 35^. For this reason, the MVD of *D. odorifera* sun leaflets was negatively correlated with LA in our research (Fig. 5c). The development of stomata and adjustment of pore aperture are regulated by complex regulatory networks that incorporate environmental cues to optimize photosynthetic capacity^36^. In previous studies, great advances in the understanding of both vein and stomatal development have been made^34, 36^, while little is known about the intersection between the pathways identified in the differentiation of these two tissues^37^.

In conclusion, we found there was positive correlation between SD and MVD in peanut plants under each light regime, and SV also remained stable under each light regime, which indicated the coordination between leaf water supply and demand. Sun leaves of *D. odorifera* and *D. renifolium* had stable SV were further confirmed. Under shading growth conditions, SV of peanut plants decreased significantly when compared to full sun conditions, which indicated the coordination change. Although MVD was positively correlated with LA, SV was independent of LA. Our findings highlight the significance of SV and provide new insight into the coordination between stomatal number and minor vein length.

## Materials and Methods

### Study site and plant material

This study was carried out in Xishuangbanna Station for Tropical Forest Ecosystem Studies (21°55′N, 101°16′E, 570 m a.s.l.), located in Xishuangbanna Tropical Botanical Garden, Chinese Academy of Sciences, Yunnan Province, SW China. Average annual temperature at the study site is 21.5 °C and average annual precipitation is 1,557 mm.

Peanut seeds were collected from different cultivated plants with the same genotype in Yishui, Shandong province (35°78′S, 118°64′E, 166 m a.s.l.) in September 2013 and stored at room temperature.

Approximately 18 seeds were sown in 18 pots (1 seed each pot) in early May 2014 in an experimental field. Pots were 15 L in size and filled with soil composed of 70% laterite and 30% washed river sand. All seeds were buried approximately 4 cm below the soil surface and the pots were placed in full sun. After germination and both cotyledons had fully emerged, 12 seedlings were randomly assigned to each of the two shade treatments^38^. These shade treatments were generated with polyethylene screening fastened to a rectangular box constructed from steel tubing that was placed over the seedlings. A quantitherm light meter/thermometer (Hansatech, England) was used to measure the irradiance under each treatment on a sunny day for three times to determine the proportion of shading. A 65% shade treatment was generated by shading with one layer of polyethylene screen netting and 96% shade was generated by two layers of polyethylene screen netting. The remaining set of the 6 potted seedlings were grown under full sun. The three light levels were used to examine whether stomatal number per minor vein length changes in different light conditions.

**Figure 7:**
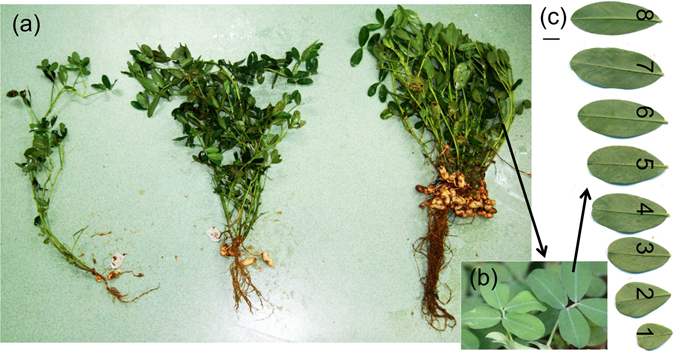
Morphology of whole peanut plants grown under three different light regimes (from left to right: 96% shade, 65% shade and full sun) (**a**). Arrangement and morphology of leaflets that make up the compound leaf of peanut (**b**). Morphology of apical leaflets taken from compound leaves located at nodes one through to eight (numbered from base to top) on the middle stem of a peanut plant grown under full sun (**c**). Scale bar is 1 cm.

After 3 months of growth, a series of mature leaves appeared on each stem of the peanut plants (Fig. 7a). According to production experience, a peanut plant is in the early stages of reproduction when it has about 10 mature leaves on the middle stem. Therefore, we collected the apical leaflet of each compound leaf from the first to eighth (numbered from bottom to top) compound leaves on the middle stem of four well-growing plants under each light treatment (Fig. 7c). The leaf node (leaf insertion levels from base to top on the same stem) was equal to the leaflet node for peanut plants.

*Dalbergia odorifera* T. Chen (*D. odorifera*) and *Desmodium renifolium* (L.) Schindl. (*D. renifolium*) grown in Xishuangbanna Tropical Botanical Garden in China were also employed in this study. We sampled four sun compound leaves from four mature *D. odorifera* trees (more than 10 years old) and numbered each leaflet from base to top along the rachis. We also sampled 12 sun leaves from one *D. renifolium* shoot from four shrubs. The four shoots were similar in age and length, and the leaves were also numbered from base to top along the shoot. Leaflet traits were measured just like those of peanut leaflets.

### Measurement of leaf traits

Leaflets were scanned with a HP scanjet G3110 and then, the area of the scanned leaflets was measured using Image J (http://rsbweb.nih.gov/ij/index.html).

Stomatal density of a leaflet was measured by the impression method. We applied clear nail varnish to a 1 cm^2^ patch on the both sides of the middle part of the leaflet surface (avoiding major veins). After 3 min, the nail polish was removed and mounted it on a glass slide to observe under the microscope (LEICA DM 2500, Germany). The stomata images were taken under 200x magnification (ca. 20 stomata in the field). Stomatal density (SD, no. mm^–2^) of each leaflet was estimated by counting the total number of stomata on both sides from 6 different fields of view on each side. Stomatal length was averaged from 12 randomly selected stomata on both sides for each leaflet.

To measure the minor vein density and thickness, approximately 1 cm^2^ section was excised from the central section of a sample leaflet which was used for measure stomata density. These leaf samples were kept in a 5% NaOH solution, which was changed weekly until the veins were exposed. The samples were washed by distilled water for 3 times, then placed on glass slides, dyed with 1% methylene blue solution, and rinsed again. For each leaf sample, six images were taken at 100x magnification using the microscope. The length and thickness of the minor veins within the view field was measured using an image analysis software (Image J). The minor vein density (MVD, mm mm^−2^) was expressed as the total length of minor veins per unit area. The stomata number per minor vein length (no. mm^−1^) was calculated by dividing SD by MVD.

### Data analyses

Statistical analyses were applied using SPSS V21 (IBM Corp. Armonk, NY, USA) and bivariate trait relationships were analyzed with Pearson’s correlation. Equal variances of the variables were tested and one-way ANOVA was used to test trait differences among the three light conditions. Two-way ANOVA was used to test the effect of leaf node position and illumination on the leaflet traits. The differences in slope or intercept of bivariate relationships between light conditions were examined in SMATR v2.0^39^.
